# Synthesis and stereochemical assignments of diastereomeric Ni(II) complexes of glycine Schiff base with (*R*)-2-(*N*-{2-[*N*-alkyl-*N*-(1-phenylethyl)amino]acetyl}amino)benzophenone; a case of configurationally stable stereogenic nitrogen

**DOI:** 10.3762/bjoc.10.41

**Published:** 2014-02-19

**Authors:** Hiroki Moriwaki, Daniel Resch, Hengguang Li, Iwao Ojima, Ryosuke Takeda, José Luis Aceña, Vadim A Soloshonok

**Affiliations:** 1 Department of Chemistry, Institute of Chemical Biology & Drug Discovery, State University of New York at Stony Brook, Stony Brook, New York 11794-3400, United States; 2 Hamari Chemicals Ltd. 1-4-29 Kunijima, Higashi-Yodogawa-ku, Osaka, Japan 533-0024; 3 Department of Organic Chemistry I, Faculty of Chemistry, University of the Basque Country, 20018 San Sebastián, Spain; 4 IKERBASQUE, Basque Foundation for Science, 48011 Bilbao, Spain

**Keywords:** amino acids, asymmetric synthesis, Ni(II) complexes, Schiff bases, stereogenic nitrogen

## Abstract

A family of chiral ligands derived from α-phenylethylamine and 2-aminobenzophenone were prepared by alkylation of the nitrogen atom. Upon reaction with glycine and a Ni(II) salt, these ligands were transformed into diastereomeric complexes, as a result of the configurational stability of the stereogenic nitrogen atom. Different diastereomeric ratios were observed depending on the substituent R introduced in the starting ligand, and stereochemical assignments were based on X-ray analysis, along with NMR studies and optical rotation measurements.

## Introduction

Since the beginning of organic chemistry, α-amino acids (α-AAs) have been an attractive target for synthetic chemists. The reported synthetic methods for assembling the RCH(NH_2_)COOH units have been explored in great detail. Structurally complex natural or tailor-made [1] α-AAs can be prepared using the currently available methodologies [2–30]; however, the chemistry of α-AAs continues to evolve [31–38]. Many of the reported synthetic methodologies have shown preference for adjusting stoichiometry or developing catalytic reactions to achieve a specific chemical and stereochemical outcome [2–30]. On the other hand, a measure of the value of a synthetic method, regardless of its stoichiometric or catalytic requirements, is the cost of the final product. In this regard, the synthetic preparation of α-AAs is far behind the biocatalytic methods that dominate their industrial production [39–40]. It was emphasized, in relatively recent reviews [39–41], that currently available, purely chemical methods for preparation of α-AAs are prohibitively expensive. The advantage of biocatalytic processes is that they can be conducted under operationally convenient conditions [42–43] and therefore are more economical. Consequently, the current emphasis in developing synthetic procedures for preparation of α-AAs is focused on simplicity of the experimental procedures and cost of the target α-AAs.

**Figure 1:**
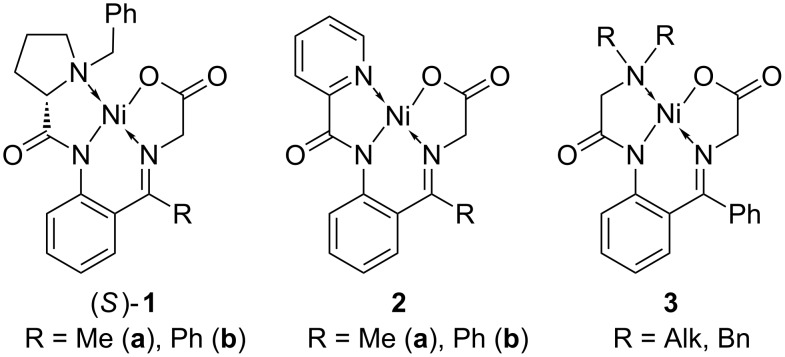
A family of chiral and achiral equivalents of nucleophilic glycine.

Among various chiral nucleophilic glycine equivalents, the Ni(II) complex of glycine Schiff base **1** (Figure 1) possesses some attractive characteristics that underscore its potential commercial application [44–47]. In particular, homologation of Ni(II) complex **1** via alkyl halide alkylation [48–50], aldol [51–53], Mannich [54–55] and Michael [56–58] addition reactions can be conducted at room temperature and without specially controlled conditions. Achiral derivatives of **1**, compounds **2** [59–60] and **3** [61–63], show even greater features of practicality and found application in the convenient synthesis of symmetrically α,α-disubstituted α-AAs [64–65], homologation under asymmetric PTC [66–67] and Michael addition reactions [68–72]. Nevertheless, the commercial application of chiral complex **1** is rather limited. The major shortcomings of compound **1** are: problematic scale up of its synthesis, partial racemization of the *N*-(benzyl)proline moiety and undesirable stereochemical outcomes. To overcome these deficiencies we initiated a project aiming to design a novel and advanced structural type of Ni(II) complexes using an inexpensive and nonracemizable chiral auxiliary. As the first step in this direction, here we describe the preparation of Ni(II) complexes of glycine Schiff base with α-phenylethylamine-derived ligands. One unusual feature of these new glycine-Ni(II) complexes is that they, along with the chiral amine residue, have a configurationally stable stereogenic nitrogen, leading to formation of two diastereomers. Consequently, stereochemical assignments of the diastereomeric products, based on crystallographic data, and sense of stereochemical preferences, were an important part of this work.

## Results and Discussion

**Scheme 1:**
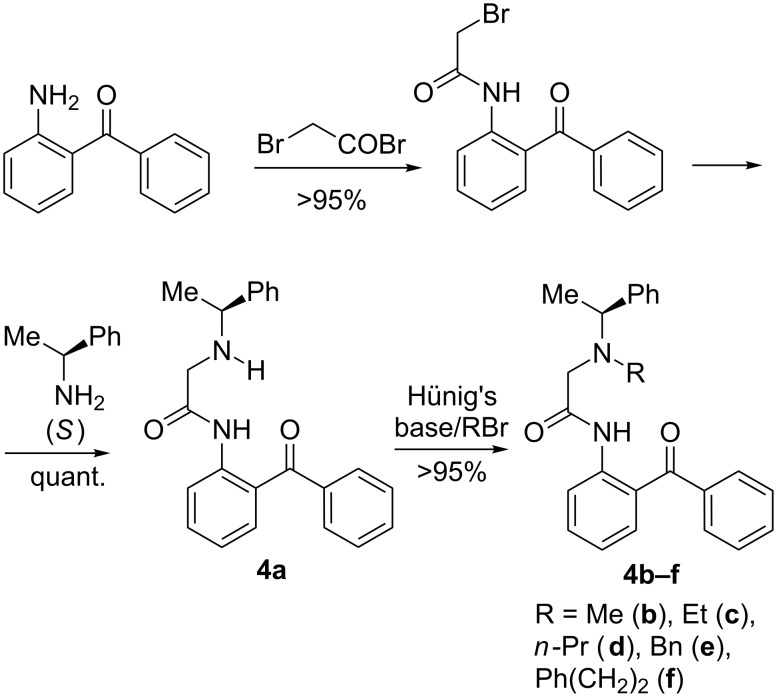
Synthesis of chiral ligands **4a–f**.

Taking advantage of the recently developed new generation of modular ligands/complexes **3** [73–74], we have designed ligands **4a**–**f** (Scheme 1) derived from α-phenylethylamine [75–76], as the source of chirality. As one can see from Scheme 1, the synthesis of ligands **4a**–**f** involves a set of very simple reactions, occurring with virtually quantitative yields [77–78].

**Scheme 2:**
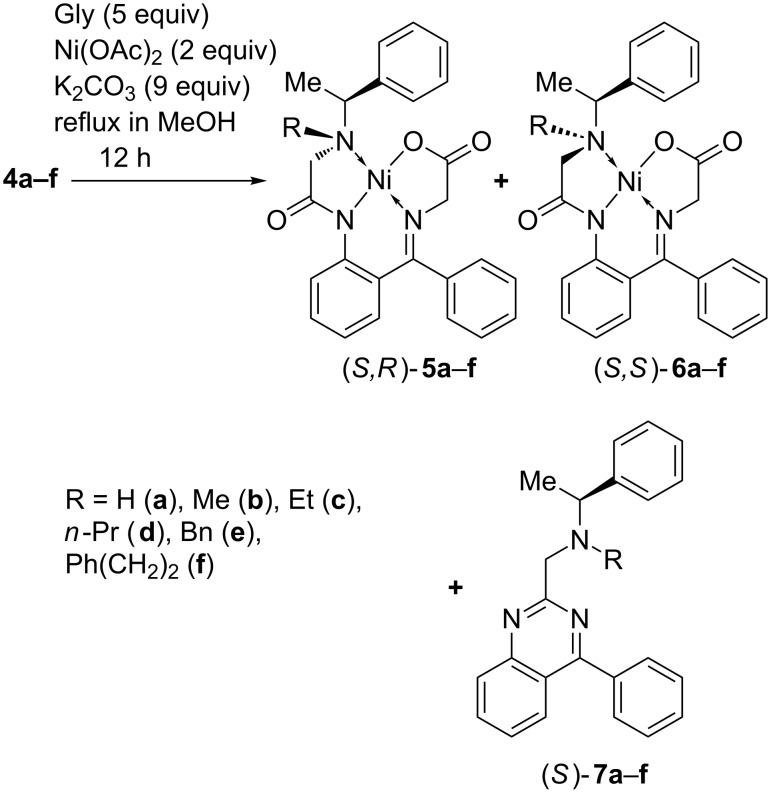
Preparation of diastereomeric Ni(II) complexes **5a**–**f** and **6a**–**f**.

To prepare the target Gly-Ni(II) complexes, ligands **4a**–**f** were heated in MeOH in the presence of glycine, Ni(OAc)_2_ and K_2_CO_3_ (Scheme 2). To compare the results, all reactions were conducted under the same conditions. The reaction mixtures were poured into water and the products were simply filtered off.

The chemical and stereochemical outcomes of the reactions, presented in Table 1, were rather unexpected. First of all, one can see a clear tendency that the increase in steric bulk of the substituent R has a significant effect on the reaction rates. In the case of R = H and Me (**4a**,**b**; Table 1, entries 1 and 2) the reactions were completed in 1 hour and the corresponding Ni(II) complexes were obtained in high yields. Under the same conditions, the reactions of Et- and *n*-Pr-containing ligands (**4c**,**d**; Table 1, entries 3 and 4) proceeded with noticeably slower rates affording the corresponding products **5** and **6** in lower yields. Still slower reaction rates were observed in the case of Bn (**4e**) and 2-(phenyl)ethyl (**4f**) bearing ligands (Table 1, entries 5 and 6). These results revealed that the steric bulk of the amine’s nitrogen plays a significant role and should be minimized to maintain practically sounding reactions rates. On the other hand, the diastereoselectivity in the formation of **5** and **6** was increasing along with the steric bulk of the substituent R, reaching the maximum of ~75/25 in the case of R = *n*-Pr and Bn groups (Table 1, entries 4 and 5). Another surprising result is that the complex(es) obtained in the reaction of NH (bearing ligand R = H) had unprecedentedly low solubility. During the complex formation reaction, the product(s) precipitated from the reaction mixture and were isolated by filtration. Solvents such as DMF, DMSO, and acetic acid were used in an attempt to dissolve the product without success. The stereochemical outcome in this reaction remains unclear.

**Table 1:** Preparation of diastereomeric Ni(II) complexes **5a**–**f** and **6a**–**f**.

Entry	R	**5**/**6** ratio^a^	Yield (%)	[α]_D_^b^	

				**5**	**6**

1	H (**a**)	N/A^c^	87.4	N/A^c^	N/A^c^
2	Me (**b**)	57/43	96.9	+88.6	+1701.8
3	Et (**c**)	61/39	67.7	−150.8	+1226.4
4	*n*-Pr (**d**)	74/26	70.1	−55.0	+1033.3
5	Bn (**e**)	76/24	31.5^d^	+303.6	+361.5
6	Ph(CH_2_)_2_ (**f**)	67/33	12.1^d^	N/A^e^	N/A^e^

^a^Determined by ^1^H NMR analysis on the crude reaction mixtures. ^b^Measured in dichloromethane, 25 ºC, *c* = ~1. ^c^The compound could not be dissolved in any solvent. ^d^The reaction was incomplete and the starting ligand was also recovered. ^e^The diastereomers could not be separated.

Since the reactions were conducted in open atmosphere, formation of byproducts (*S*)-**7a**–**f** (5–20%) were observed in all cases. Compounds (*S*)-**7a**–**f** result from the reaction of atmospheric oxygen with the corresponding enolates derived from glycine complexes **5** and **6** and the basic reaction conditions [79–80]. Formation of 4-phenylquinazoline derivatives (*S*)-**7a**–**f** can be prevented by conducting the reactions in oxygen-free atmosphere. Another general trend is apparent when considering the observed optical rotations of the diastereomeric products **5** and **6**. In all cases, the major diastereomers **5** had low-magnitude rotations as compared with high-magnitude dextrorotatory rotations of **6**. This trend can be used to assign the configurations of products **5** and **6** and related compounds. It is interesting to note that the difference in optical rotation between diastereomers **5e** and **6e** was the least pronounced in the series. Most likely, some similarity in the structure of *N*-benzyl and *N*-α-(methyl)benzyl groups results in low impact on the optical rotation of diastereomers **5e** and **6e**.

**Figure 2:**
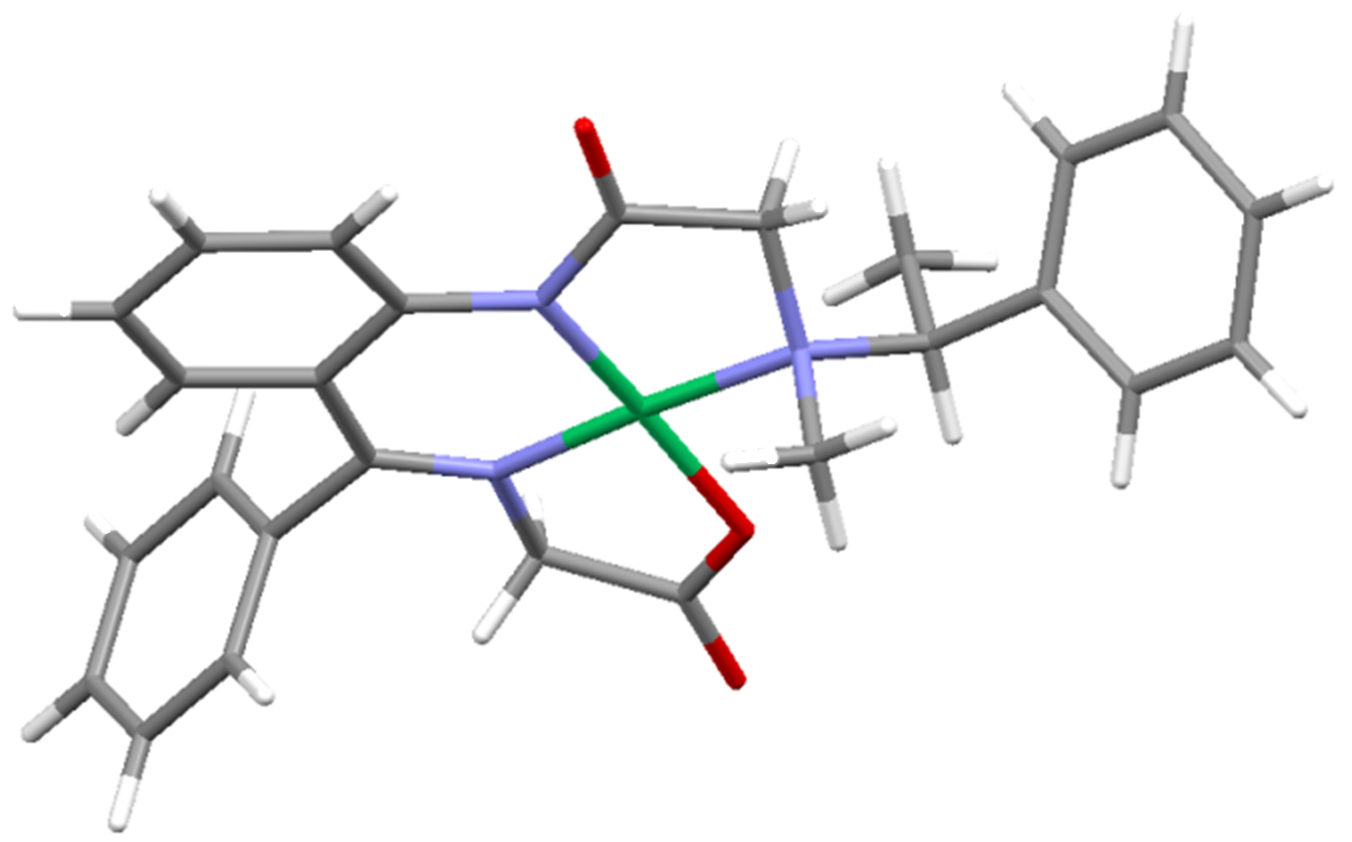
Crystallographic structure of (*S*_C_*R*_N_)-**5b**.

Knowledge of one of the stereocenters in diastereomers **5** and **6** allowed us to use X-ray crystallography to determine the absolute configuration of the major product **5b** [81]. The structure presented in Figure 2 shows its (*S*_C_*R*_N_) absolute configuration. Accordingly, the second product, diastereomer **6b**, has (*S*_C_*S*_N_) configuration. The X-ray structure of **5b** revealed the complex is not fully planar as expected. The benzophenone chelate ring system deviates from planarity by 14.4° (0.16 e.s.d.). The torsion angle of Ni2–O5–C10–C29 is −10.9° further indicating a deviation from planarity. One of the rings in the benzophenone chelate is rotated 85.8° (0.36 e.s.d.) out of the plane presumably due to sterics. This rotation minimizes the Ar–H···H–Ar repulsion as well as repulsion with methylene group of the glycine moiety and the Ar–H.

**Figure 3:**
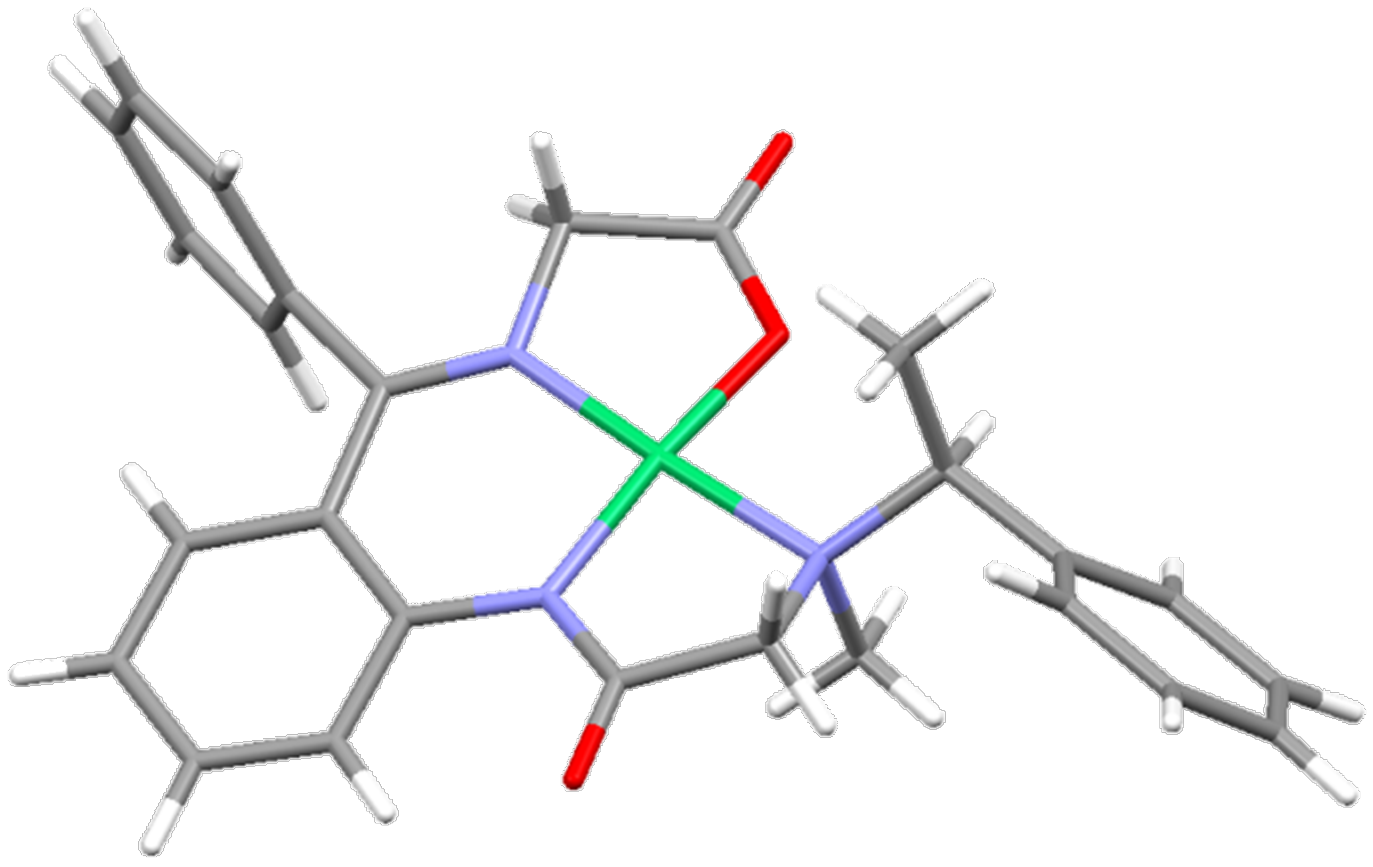
Crystallographic structure of (*S*_C_*R*_N_)-**5b** showing an exposure of the methyl moiety of α-phenylethylamine to the Ni(II).

As we have mentioned above, there is a great degree of similarity in the chiroptical properties of complexes **5b**–**e** and **6b**–**e**, sufficient to make the corresponding stereochemical assignments. Moreover, there is also another general trend observed in the ^1^H NMR spectra of compounds **5** and **6**. In particular, as it is evident form Figure 3, the α-phenylethylamine moiety's methyl group is located in relatively close proximity to the Ni(II) atom, causing the methyl protons to shift more downfield [82–85]. Accordingly, in all ^1^H NMR spectra of compounds **5b**–**e** this methyl group is shifted downfield (2.5–2.9 ppm) as compared with the chemical shift of the same methyl in diastereomers **6b**–**e** (2.2–1.75 ppm).

Combining together the crystallographic, chiroptical and ^1^H NMR data of compounds **5** and **6**, we can confidently assign the (*S*_C_*R*_N_) absolute configuration to all products **5b–e** and (*S*_C_*S*_N_) stereochemistry to the complexes **6b**–**e**.

## Conclusion

In conclusion, this exploratory work has revealed that: 1) Application of α-phenylethylamine as the source of stereochemistry information in the novel Ni(II) complexes, results in the formation of diastereomeric glycine Schiff base products due to the configurational stability of the stereogenic nitrogen. 2) The steric bulk of the substituent R on the α-phenylethylamine residue has a negative effect on the reaction rates and a positive influence on the stereochemical outcome. 3) The optimal size of the R group is *n*-Pr or Bn. 4) Synthesis of this type of Ni(II) complexes should be conducted under oxygen-free conditions to avoid the formation of byproducts, resulting from the oxidation of the corresponding enolates. 5) The (*S*_C_*R*_N_) stereochemistry of the Ni(II) complexes is generally preferred. Drawing from these results, an improved design of new ligands is currently under investigation in our laboratories.

## Experimental

### General procedure for the preparation of ligands **4b–f**

In analogous manner as previously reported [77], secondary amine **4a** [78] (1 equiv), *N,N*-diisopropylethylamine (1.5 equiv), alkyl halide (1.5–3.5 equiv) and 10 mL of MeCN were placed in a round bottom flask and stirred at room temperature under nitrogen. After completion of the reaction (monitored by TLC), the reaction mixture was evaporated to dryness under vacuum. The residue was dissolved in 5 mL of CH_2_Cl_2_ and washed with 5 mL of water. The aqueous layer was washed with 3 × 5 mL fractions of CH_2_Cl_2_. The collected organic fractions were dried over MgSO_4_ and the solvent was removed under vacuum to yield the crude product. Analytically pure samples of **4b–f** were obtained by column chromatography (Hex/EtOAc).

**(*S*)-*N*-(2-benzoylphenyl)-2-(methyl-(1-phenylethyl)amino)acetamide (4b):** From **4a** (6.14 g, 17.23 mmol) and MeI (4.3 mL, 68.52 mmol), 4.61 g (72.4%) of **4b**. White solid. Mp 80–82 °C; [α]_D_^25^ +23.7 (*c* 1.55, CHCl_3_); ^1^H NMR (300 MHz, CDCl_3_) δ 1.54 (d, *J* = 6.9 Hz, 3H), 2.44 (s, 3H), 3.12 (d, *J* = 16.8 Hz, 1H), 3.25 (d, *J* = 16.8 Hz, 1H), 3.82 (q, *J* = 6.6 Hz, 1H), 6.87–6.92 (m, 1H), 7.15–7.22 (m, 1H), 7.25–7.28 (m, 2H), 7.50–7.72 (m, 7H), 7.73–7.92 (m, 2H), 8.69 (d, *J* = 8.7 Hz, 1H), 11.67 (br, 1H); ^13^C NMR (75 MHz, CDCl_3_) δ 18.4, 40.8, 58.7, 63.5, 121.5, 122.1, 125.1, 127.1, 127.6, 128.2, 130.0, 132.3, 132.5, 133.2, 138.4, 139.0, 142.8, 171.1, 197.9; HRMS: [M + H]^+^ calcd for C_24_H_25_N_2_O_2_, 373.1916; found, 373.1923.

### General procedure for the preparation of Ni(II) complexes **5b–f** and **6b–f**

To a flask containing a methanol solution of ligand **4b–f** (1 equiv), Ni(OAc)_2_·4H_2_O (2 equiv) and glycine (5.0 equiv), was added K_2_CO_3_ (9 equiv), and the reaction mixture was stirred at 60–70 °C. The progress of the reaction was monitored by TLC and upon completion (consumption of the reagent **4**), the reaction mixture was poured into ice water. The target product was extracted three times with CH_2_Cl_2_. The combined organic layer was dried over anhydrous MgSO_4_ and evaporated under vacuum. After evaporation of the solvents, the target complexes **5b–f** and **6b–f** were obtained in diastereomerically pure form by separation on silica-gel column chromatography.

**5b and 6b**: From **4b** (512 mg, 1.375 mmol), 647.3 mg (96.9 %) of a 57:43 mixture of **5b** and **6b**. Data of **5b**: Red solid. Mp 265–267 °C; [α]_D_^25^ +88.6 (*c* 1.0, CH_2_Cl_2_); ^1^H NMR (300 MHz, CDCl_3_) δ 2.49 (d, *J* = 6.9 Hz, 3H), 2.57 (d, *J* = 16.8 Hz, 1H), 2.83 (s, 3H), 3.65–3.85 (m, 2H), 3.90–4.00 (m, 1H), 3.97 (d, *J* =16.8 Hz, 1H), 6.77–7.05 (m, 4H), 7.26–7.40 (m, 6H), 7.51–7.53 (*m*, 3H), 8.63 (d, *J* = 9.0 Hz, 1H). ^13^C NMR (75 MHz, CDCl_3_): 19.7, 47.5, 58.9, 61.3, 64.6, 121.1, 124.2, 125.1, 125.8, 126.0, 128.5, 129.0, 129.5, 129.6, 130.1, 132.7, 133.5, 134.1, 134.6, 142.5, 172.0, 177.2, 178.5. HRMS: [M + H]^+^ calcd for C_26_H_26_N_3_O_3_Ni, 486.1328; found, 486.1335. Data of **6b**: Red solid. Mp 269–272 °C. [α]_D_^25^ +1701.8 (*c* 1.0, CH_2_Cl_2_); ^1^H NMR (300 MHz, CDCl_3_) δ 2.27 (s, 3H), 2.34 (d, *J* = 6.9 Hz, 3H), 3.35 (d, *J* = 16.5 Hz, 1H), 3.60–3.72 (m, 3H), 4.32 (q, *J* = 6.9 Hz, 1H), 6.75–6.93 (m, 3H), 7.13–7.18 (m, 1H), 7.30–7.51 (m, 9H), 8.48 (d, *J* = 8.1 Hz, 1H); ^13^C NMR (75 MHz, CDCl_3_) δ 17.6, 42.2, 61.1, 62.2, 66.2, 121.3, 124.3, 125.5, 125.6, 126.2, 128.7, 129.1, 129.3, 129.7, 129.9, 130.2, 132.5, 133.4, 134.5, 134.8, 142.3, 171.9, 176.0, 177.2. HRMS: [M + H]^+^ calcd for C_26_H_26_N_3_O_3_Ni, 486.1328; found, 486.1337.

## Supporting Information

File 1 Experimental data for compounds **4c–f**, **5c–e** and **6c–e**.
