# Supplementary material for: Synthesis and stereochemical assignments of diastereomeric Ni(II) complexes of glycine Schiff base with (R)-2-(N-{2-[N-alkyl-N-(1-phenylethyl)amino]acetyl}amino)benzophenone; a case of configurationally stable stereogenic nitrogen
Source: Beilstein J Org Chem. 2014 Feb 19;10:442–8. doi: 10.3762/bjoc.10.41 (PMC3943998; doi:10.3762/bjoc.10.41)
Supplement: File 1 — Experimental data for compounds 4c–f, 5c–e and 6c–e. [file Beilstein_J_Org_Chem-10-442-s001.pdf]

Supporting Information

for

Synthesis and stereochemical assignments of diastereomeric Ni(II) complexes of glycine Schiff base with (*R*)-2-(*N*-{2-[*N*-alkyl-*N*-(1-phenylethyl)amino]acetyl}amino)benzophenone; a case of configurationally stable stereogenic nitrogen

Hiroki Moriwaki*[1,2], Daniel Resch[1], Hengguang Li[1], Iwao Ojima[1], Ryosuke Takeda[2], José Luis Aceña[3] and Vadim A. Soloshonok*[3,4]

Address: [1]Department of Chemistry, Institute of Chemical Biology & Drug Discovery, State University of New York at Stony Brook, Stony Brook, New York 11794-3400, United States, [2]Hamari Chemicals Ltd. 1-4-29 Kunijima, Higashi-Yodogawa-ku, Osaka, Japan 533-0024, [3]Department of Organic Chemistry I, Faculty of Chemistry, University of the Basque Country, 20018 San Sebastián, Spain and [4]IKERBASQUE, Basque Foundation for Science, 48011 Bilbao, Spain

Email: Vadim A. Soloshonok - vadym.soloshonok@ehu.es

*Corresponding author

Experimental data for compounds 4c–f, 5c–e and 6c–e.

(*S*)-*N*-(2-benzoylphenyl)-2-(ethyl-(1-phenylethyl)amino)acetamide (4c): From **4a** (2.58 g, 7.20 mmol) and EtI (2.31 mL, 28.8 mmol), 1.0 g (35.9%) of **4c**. Colorless oil; $[\alpha]_D^{25}$= +36.0 (*c* = 1.25, $CHCl_3$). ^{1}H-NMR (300 MHz, $CDCl_3$): 1.16 (t, *J* = 7.1 Hz, 3H), 1.44 (d, *J* = 6.8 Hz, 3H), 2.56–2.69 (m, 1H), 2.72–2.85 (m, 1H), 3.16 (d, *J* = 17.4 Hz, 1H), 3.26 (d, *J* = 17.3 Hz, 1H), 4.02 (q, *J* = 6.7 Hz, 1H), 7.12 (td, *J* = 7.6, 1.0 Hz, 1H), 7.16–7.20 (m, 3H), 7.44–7.49 (m, 2H), 7.50–7.60 (m, 4H), 7.66 (tt, *J* = 7.4, 1.3 Hz, 1H), 7.86 (d, *J* = 7.5 Hz, 2H), 8.65 (d, *J* = 8.4 Hz, 1H), 11.54 (br, 1H). ^{13}C-NMR (75 MHz, $CDCl_3$): 11.9, 17.1, 46.8, 55.1, 59.9, 121.2, 121.8, 125.0, 126.8, 127.6, 127.9, 128.1, 129.9, 131.9, 132.4, 132.9, 138.2, 138.7, 142.5, 171.9, 197.5. HRMS: calcd for $C_{25}H_{27}N_2O_2$ $[M + H]^+$ 387.2073, found 387.2087.

(*S*)-*N*-(2-benzoylphenyl)-2-((1-phenylethyl)(propyl)amino)acetamide (4d): From **4a** (3.68 g, 10.25 mmol) and *n*-PrBr (1.4 mL, 15.38 mmol), 1.01 g (15.1%) of **4d**. Colorless oil. $[\alpha]_D^{25}$= +28.8 (*c* = 1.02, $CHCl_3$). ^{1}H-NMR (300 MHz, $CDCl_3$): 0.81 (t, *J* = 7.5 Hz, 3H), 1.42 (d, *J* = 6.6 Hz, 3H), 1.61 (sext, *J* = 8.1 Hz, 2H), 2.40–2.55 (m, 1H), 2.58–2.70 (m, 1H), 3.14 (d, *J* = 17.4 Hz, 1H), 3.25 (d, *J* = 17.4 Hz, 1H), 3.98 (q, *J* = 6.6 Hz, 1H), 7.07–7.17 (m, 4H), 7.42–7.54 (m, 6H), 7.60–7.63 (m, 1H), 7.82–7.86 (m, 2H), 8.64 (dd, *J* = 8.7, 0.9 Hz, 1H), 11.45 (br, 1H). ^{13}C-NMR (75 MHz, $CDCl_3$): 11.6, 17.1, 20.3, 55.3, 55.8, 60.5, 121.4, 121.9, 125.3, 126.9, 127.8, 128.0, 128.1, 130.0, 131.9, 132.5, 133.0, 138.2, 138.7, 142.4, 172.0, 197.6. HRMS: calcd for $C_{26}H_{29}N_2O_2$ $[M + H]^+$ 401.2229, found 401.2233.

(*S*)-*N*-(2-benzoylphenyl)-2-(benzyl-(1-phenylethyl)amino)acetamide (4e): From **4a** (2.61 g, 7.28 mmol) and BnBr (1.32 mL, 10.92 mmol), 3.0 g (91.7%) of **4e**. Colorless oil; $[\alpha]_D^{25}$= +28.1 (*c* = 3.81, $CHCl_3$). ^{1}H-NMR (300 MHz, $CDCl_3$): 1.43 (d, *J* = 6.9 Hz, 3H), 1.47 (s, 1H), 3.02 (d, *J* = 16.8 Hz, 1H), 3.22 (d, *J* =

16.8 Hz, 1H), 3.36 (d, *J* = 12.9 Hz, 1H), 3.72 (d, *J* = 13.2 Hz, 1H), 3.84 (q, *J* = 7.2 Hz, 1H), 6.96–7.01 (m, 4H), 7.13–7.20 (m, 3H), 7.37–7.48 (m, 8H), 7.55–7.57 (m, 1H), 7.78–7.81 (m, 2H), 8.44 (dd, *J* = 7.5, 1.2 Hz, 1H), 11.22 (br, 1H). ^{13}C-NMR (75 MHz, $CDCl_3$): 17.1, 55.4, 56.9, 60.8, 121.3, 121.8, 125.1, 127.0, 127.9, 128.1, 128.1, 129.3, 130.1, 131.8, 132.6, 132.9, 137.8, 138.1, 138.7, 141.4, 171.4, 197.6. HRMS: calcd for $C_{30}H_{29}N_2O_2$ $[M + H]^+$ 449.2229, found 449.2250.

(*S*)-*N*-(2-benzoylphenyl)-2-(phenethyl-(1-phenylethyl)amino)acetamide (4f): From **4a** (3.07 g, 7.36 mmol) and $Ph(CH_2)_2Br$ (1.76 mL, 12.85 mmol), 505 mg (14.8%) of **4f**. Colorless oil; $[\alpha]_D^{25}$= +6.1 (*c* = 0.59, $CHCl_3$). ^{1}H-NMR (300 MHz, $CDCl_3$): 1.46 (d, *J* = 6.3 Hz, 3H), 2.72–2.93 (m, 3H), 3.22 (d, *J* = 17.1 Hz, 1H), 3.32 (d, *J* = 17.1 Hz, 1H), 4.09 (q, *J* = 6.9 Hz, 1H), 6.99–7.09 (m, 2H), 7.10–7.37 (m, 9H), 7.38–7.46 (m, 3H), 7.47–7.62 (m, 8H), 8.60 (dd, *J* = 9.6, 2.1 Hz, 1H), 11.55 (br, 1H). ^{13}C-NMR (75 MHz, $CDCl_3$): 17.3, 33.7, 55.2, 55.8, 60.4, 121.5, 122.1, 125.3, 125.9, 127.2, 127.9, 128.2, 128.3, 128.5, 130.1, 132.2, 132.6, 133.2, 138.4, 138.8, 139.7, 142.3, 171.8, 197.8. HRMS: calcd for $C_{31}H_{31}N_2O_2$ $[M + H]^+$ 463.2386, found 463.2398.

5c and 6c: From **4c** (240 mg, 0.62 mmol), 210 mg (67.7 %) of a 61:39 mixture of **5c** and **6c**. Data of **5c**: Red solid. M.p. 207-210 ºC. $[\alpha]_D^{25}$= –150.8 (*c* = 1.0, CH_2Cl_2). ^{1}H-NMR (300 MHz, $CDCl_3$): 2.05–2.20 (m, 1H), 2.41 (t, *J* = 6.9 Hz, 3H), 2.59 (d, *J* = 17.1 Hz, 1H), 2.79 (d, *J* = 6.6 Hz, 3H), 2.85–2.95 (m, 1H), 3.76 (d, *J* = 6.0 Hz, 2H), 3.95 (d, *J* =17.1 Hz, 1H), 4.14 (q, *J* = 6.9 Hz, 1H), 6.77–6.90 (m, 2H), 7.06–7.09 (m, 2H), 7.31–7.34 (m, 6H), 7.35–7.54 (m, 3H), 8.63 (dd, *J* = 8.7, 1.2 Hz, 1H). ^{13}C-NMR (75 MHz, $CDCl_3$): 14.0, 20.8, 55.0, 56.5, 61.3, 64.2, 121.0, 124.2, 125.1, 125.9, 126.0, 128.5, 128.9, 129.5, 129.6, 129.8, 132.6, 133.5, 134.7, 134.9, 142.7, 171.8, 177.2, 180.0. HRMS: calcd for $C_{27}H_{28}N_3O_3Ni$

[M + H]$^+$ 500.1484, found 500.1485. Data of **6c**: Red solid. M.p. 210-213 ºC. $[\alpha]_D^{25}$= +1226.4 (*c* = 1.0, CH_2Cl_2). ^{1}H-NMR (300 MHz, $CDCl_3$): 1.78 (d, *J* = 6.9 Hz, 3H), 2.05–2.20 (m, 1H), 2.36 (t, *J* = 6.9 Hz, 3H), 2.85–2.95 (m, 1H), 3.35 (d, *J* = 17.4 Hz, 1H), 3.48 (d, *J* = 17.1 Hz, 1H), 3.64 (d, *J* = 19.8 Hz, 1H), 3.75 (d, *J* = 20.1 Hz, 1H), 4.50 (q, *J* = 7.2 Hz, 1H), 6.68–6.75 (m, 2H), 6.94–7.06 (m, 2H), 7.19–7.51 (m, 6H), 8.06 (d, *J* = 7.2 Hz, 2H), 8.35 (d, *J* = 7.8 Hz, 1H). ^{13}C-NMR (75 MHz, $CDCl_3$): 13.6, 14.1, 50.6, 55.2, 61.0, 66.7, 120.8, 124.2, 125.1, 125.8, 126.1, 128.9, 129.2, 129.4, 129.6, 129.7, 130.0, 132.2, 133.1, 134.7, 138.7, 142.4, 171.5, 177.2, 179.1. HRMS: calcd for $C_{27}H_{28}N_3O_3Ni$ [M + H]$^+$ 500.1484, found 500.1493.

5d and 6d: From **4d** (1.0 g, 2.50 mmol), 901 mg (70.1 %) of a 74:26 mixture of **5d** and **6d**. Data of **5d**: Red solid. M.p. 155-157 ºC. $[\alpha]_D^{25}$= –55.0 (*c* = 1.0, CH_2Cl_2). ^{1}H-NMR (300 MHz, $CDCl_3$): 1.10 (t, *J* = 7.5 Hz, 3H), 1.98 (td, *J* = 4.2, 1.5 Hz, 1H), 2.45–2.60 (m, 1H), 2.56 (d, *J* = 17.1 Hz, 1H), 2.73 (td, *J* = 4.2, 1.5 Hz, 1H), 2.80 (d, *J* = 6.9 Hz, 3H), 3.76 (d, *J* = 7.8 Hz, 2H), 3.98 (d, *J* = 17.1 Hz, 1H), 4.05–4.15 (m, 1H), 4.13 (q, *J* = 6.9 Hz, 1H), 6.74–6.80 (m, 1H), 6.82–6.91 (dd, *J* = 8.4, 1.8 Hz, 1H), 7.05–7.10 (m, 2H), 7.29–7.37 (m, 5H), 7.50–7.55 (m, 3H), 8.65 (dd, *J* = 8.7, 0.9 Hz, 1H). ^{13}C-NMR (75 MHz, $CDCl_3$): 11.8, 20.7, 22.2, 56.9, 61.2, 62.5, 64.1, 120.9, 124.1, 125.0, 125.8, 125.9, 128.4, 128.9, 129.4, 129.5, 129.8, 132.5, 133.4, 134.6, 134.8, 142.6, 171.7, 177.2, 179.9. HRMS: calcd for $C_{28}H_{30}N_3O_3Ni$ [M + H]$^+$ 514.1641, found 514.1642. Data of **6d**: Red solid. M.p. 156-158 ºC. $[\alpha]_D^{25}$= +1033.3 (*c* = 1.0, CH_2Cl_2). ^{1}H-NMR (300 MHz, $CDCl_3$): 1.15 (t, *J* = 7.5 Hz, 3H), 1.72 (d, *J* = 6.9 Hz, 3H), 1.99 (td, *J* = 4.2, 1.5 Hz, 1 H), 2.30–2.45 (m, 1 H), 2.79 (td, *J* = 4.2, 1.5 Hz, 1H), 3.30 (d, *J* = 17.1 Hz, 1H), 3.55 (d, *J* = 17.1 Hz, 1H), 3.68 (d, *J* = 20.1 Hz, 2H), 4.05–4.20 (m, 1H), 6.64–6.92 (m, 1H), 4.65 (q, *J* = 6.9 Hz, 1H), 6.60–6.80 (m, 2H), 6.96–7.00 (m,

1H), 7.16–7.22 (m, 1H), 7.30–7.35 (m, 1H), 7.45–7.51 (m, 4H), 8.06 (d, *J* = 6.9 Hz, 1H), 8.35 (dd, *J* = 8.7, 0.9 Hz, 1H). ^{13}C-NMR (75 MHz, $CDCl_3$): 12.0, 13.7, 22.1, 55.9, 58.6, 61.1, 66.4, 120.7, 124.1, 125.0, 125.8, 126.0, 128.9, 129.2, 129.4, 129.6, 129.6, 130.0, 132.2, 133.1, 134.6, 138.9, 142.4, 171.4, 177.1, 179.3. HRMS: calcd for $C_{28}H_{30}N_3O_3Ni$ $[M + H]^+$ 514.1641, found 514.1649.

5e and 6e: From **4e** (1.0 g, 2.23 mmol), 287 mg (31.5 %) of a 76:24 mixture of **5e** and **6e**. Data of **5e**: Red solid. M.p. 136-138 ºC. $[\alpha]_D^{25}$= +303.6 (*c* = 1.0, CH_2Cl_2). ^{1}H-NMR (300 MHz, $CDCl_3$): 2.81 (d, *J* = 17.4 Hz, 1H), 2.89 (d, *J* = 6.9 Hz, 3H), 2.95 (d, *J* = 12.0 Hz, 1H), 3.72 (d, *J* = 20.1 Hz, 1H), 3.85 (d, *J* = 19.8 Hz, 1H), 4.01 (d, *J* = 17.1 Hz, 1H), 4.27 (t, *J* = 7.2 Hz, 1H), 4.29 (d, *J* =10.5 Hz, 1H), 6.63–6.75 (m, 2H), 7.02–7.27 (m, 4H), 7.38–7.53 (m, 10H), 8.11 (d, *J* = 8.1 Hz, 1H), 8.32 (*d*, *J* = 8.4 Hz, 2H). ^{13}C-NMR (75 MHz, $CDCl_3$): 20.7, 56.4, 61.2, 65.1, 65.6, 120.6, 124.1, 124.8, 125.8, 126.1, 128.6, 128.9, 129.1, 129.2, 129.4, 129.6, 130.1, 132.1, 132.3, 132.9, 134.6, 134.8, 142.2, 171.5, 177.2, 179.3. HRMS: calcd for $C_{32}H_{30}N_3O_3Ni$ $[M + H]^+$ 562.1641, found 562.1639. Data of **6e**: Red solid. M.p. 142-145 ºC. $[\alpha]_D^{25}$= +361.5 (*c* = 1.0, CH_2Cl_2). ^{1}H-NMR (300 MHz, $CDCl_3$): 2.82 (d, *J* = 17.1 Hz, 1H), 2.89 (d, *J* = 6.9 Hz, 3H), 2.95 (d, *J* = 12.0 Hz, 1H), 3.72 (d, *J* = 20.1 Hz, 1H), 3.85 (d, *J* = 20.1 Hz, 1H), 4.01 (d, *J* = 17.4 Hz, 1H), 4.27 (t, *J* = 7.2 Hz, 1H), 4.30 (d, *J* =11.1 Hz, 1H), 6.64–7.11 (m, 6H), 7.38–7.53 (m, 12H), 8.11 (d, *J* = 8.7 Hz, 1H), 8.32 (d, *J* = 6.9 Hz, 2H). ^{13}C-NMR (75 MHz, $CDCl_3$): 20.7, 56.4, 61.2, 65.2, 65.7, 120.6, 124.2, 124.9, 125.8, 126.1, 128.7, 129.0, 129.2, 129.2, 129.4, 129.6, 130.1, 132.1, 132.9, 134.6, 134.8, 142.3, 171.5, 177.3, 179.3. HRMS: calcd for $C_{32}H_{30}N_3O_3Ni$ $[M + H]^+$ 562.1641, found 562.1638.
